# Impact of an analytical intervention to assure the accuracy of LIAISON QuantiFERON-TB Gold Plus results

**DOI:** 10.1128/jcm.01879-24

**Published:** 2025-01-28

**Authors:** Karolina Pusz-Bochenska, Niaz Banaei

**Affiliations:** 1 Department of Pathology, Stanford University School of Medicine 158566 , Stanford, California, USA; 2 Division of Infectious Diseases & Geographic Medicine, Stanford University School of Medicine 196261 , Stanford, California, USA; 3 Clinical Microbiology Laboratory, Stanford Health Care 474436 , Palo Alto, California, USA; University of Manitoba, Winnipeg, Manitoba, Canada

**Keywords:** QuantiFERON, LIAISON, ELISA, QFT-Plus, IGRA, *Mycobacterium tuberculosis*

## LETTER

Interferon-gamma (IFN-γ) release assays such as QuantiFERON-TB Gold Plus (QFT-Plus; Qiagen, Hilden, Germany) are utilized to screen for *Mycobacterium tuberculosis* infection (1, 2). To enhance efficiency and address staffing shortages, some laboratories have switched from semi-automated ELISA instruments to the fully automated Liaison XL chemiluminescence immunoassay (CLIA) for the measurement of IFN-γ in the QFT-Plus tubes (3). However, prior studies have shown CLIA inflates the IFN-γ response which results in false-positive results in patients with borderline-negative results (4–7). We have previously shown that confirming borderline-positive results (TB1-Nil and or TB2-Nil 0.35–1.0 IU/mL) with ELISA can serve to mitigate the majority of false-positive results with CLIA (4, 8). In addition, we have shown that discordant qualitative results between TB1-Nil and TB2-Nil could suggest false-positive QFT-Plus results (9). Thus, before implementing CLIA for clinical testing at our institution, we made it our laboratory policy to confirm borderline-positive CLIA results with ELISA and to repeat high-positive discordant results (TB1-Nil or TB2-Nil > 1.0 IU/mL and the other tube <0.35 IU/mL) with CLIA to mitigate reporting false-positive results. This study aimed to assess the impact of this algorithm on the QFT-Plus positivity rate and false-positive reporting at our institution.

A total of 38,224 QFT-Plus tests were performed with CLIA at Stanford Health Care clinic laboratories from September 2023 to October 2024. After initial testing with CLIA, the positivity rate was 6.1% (2,333) and the indeterminate rate was 0.6% (220). After retesting 1,528 (4.0%) samples with borderline positive (1402) and high-positive discordant TB1-Nil/TB2-Nil (126) results, the reported positivity rate was 3.4% (1,311; *P* < 0.001) and the indeterminate rate was 0.6% (243). By repeating testing, 42.8% (999/2,333) of all initial positives with CLIA were reported as negative.

**Fig 1:**
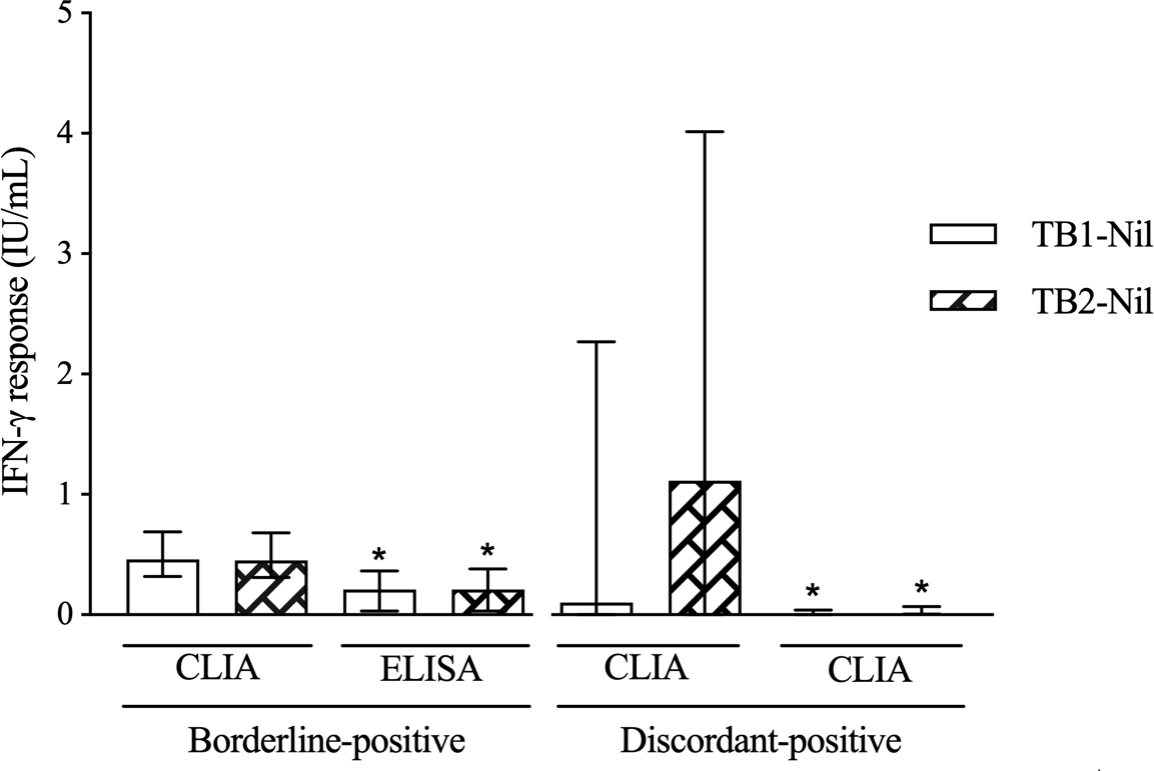
Initial and repeat QFT-Plus results for borderline-positive and high discordant-positive TB1/TB2 QFT-Plus results with Liaison CLIA. Borderline-positive Liaison CLIA result was defined as TB1-Nil and or TB2-Nil response ≥0.35 and ≤1 IU/mL. High discordant-positive Liaison CLIA result was defined as TB1-Nil or TB2-Nil response >1 IU/mL and the response in the other tube <0.35 IU/mL. Initial CLIA results are shown on the left for discordant positives. Bars show medians and whiskers show the interquartile range. * indicates statistical significance for comparison of repeat results to initial results using the Mann-Whitney test.

Upon retesting 1,402 (3.7%) borderline positives with ELISA, 35.3% (495) remained positive, 63.1% (884) tested negative, and 1.7% (23) changed to indeterminate. The median IFN-γ response for TB1-Nil and TB2-Nil was 0.46 IU/mL and 0.45 IU/mL, respectively, with initial CLIA testing and 0.21 IU/mL (*P* < 0.001) and 0.21 IU/mL (*P* < 0.001) after retesting with ELISA (Table 1; Fig. 1).

**TABLE 1:** Initial and repeat results for borderline-positive and high discordant-positive TB1/TB2 QFT-Plus results with Liaison CLIA^*a*^

	Borderline-positive Liaison CLIA results retested with ELISA (*n* = 1,402)				High discordant-positive Liaison CLIA results retested with CLIA (*n* = 126)			
	Liaison CLIA		ELISA		Liaison CLIA		Liaison CLIA	
	TB1-NIL	TB2-Nil	TB1-Nil	TB2-Nil	TB1-Nil	TB2-Nil	TB1-Nil	TB2-Nil
Median	0.46	0.45	0.21 (*P* < 0.001)	0.21 (*P* < 0.001)	0.1	1.12	0 (*P* < 0.001)	0 (*P* < 0.001)
Range	−8.16 to 9.96	−8.73 to 10.0	−0.31 to 4.31	−0.2 to 5.60	−4.98 to 9.98	−4.27 to 10.0	−3.08 to 7.84	−3.22 to 6.34
IQR	0.32 to 0.69	0.31–0.68	0.03 to 0.36	0.03 to 0.38	−0.09 to 2.27	−0.01 to 4.01	−0.02 to 0.04	−0.01 to 0.63

^*a*^ Borderline-positive Liaison CLIA result was defined as TB1-Nil and or TB2-Nil response ≥0.35 and ≤1 IU/mL. High discordant-positive Liaison CLIA result was defined as TB1-Nil or TB2-Nil response >1 IU/mL and the response in the other tube <0.35 IU/mL. *P* values are for comparison of initial result to retest result using the Mann Whitney test. IQR, interquartile range.

The 126 (0.3%) high-positive discordant results were due to positive TB2-Nil in 69 (54.8%) tests. Upon retesting with CLIA, 115 (91.3%) changed to negative and 11 (8.7%) remained positive. The median IFN-γ response for TB1-Nil and TB2-Nil was 0.1 IU/mL and 1.12 IU/mL, respectively, with initial CLIA testing and 0 IU/mL (*P* < 0.001) and 0 IU/mL (*P* < 0.001), respectively, after retesting with CLIA (Table 1; Fig. 1).

The findings from this study are consistent with prior studies on the inflated QFT-Plus responses with CLIA (4–7) and show that the problem we encountered during our validation phase persisted during clinical implementation (4, 8). Importantly, 63.1% of borderline positives retested with ELISA were reported as negative. In addition, 91.3% of high-positive discordant results repeated with CLIA were reported negative. During the first 13 months of clinical testing using CLIA, we avoided reporting 999 false-positive QFT-Plus results. Our findings underscore the need for clinical laboratories to adopt an analytical algorithm to mitigate reporting false-positive results with CLIA at the cost of repeating about 4.0% of all QFT-Plus tests. Such a diagnostic stewardship measure is essential to prevent unnecessary clinical workups performed to rule out active tuberculosis and to avoid prophylaxis therapy.
